# Supplementary material for: Deciphering Gorilla gorilla gorilla immunoglobulin loci in multiple genome assemblies and enrichment of IMGT resources
Source: Front Immunol. 2024 Oct 10;15:1475003. doi: 10.3389/fimmu.2024.1475003 (PMC11499206; doi:10.3389/fimmu.2024.1475003)

Legend

- V-GENE functionnal
- V-GENE ORF
- V-GENE pseudogene
- J-GENE functionnal
- J-GENE ORF
- J-GENE pseudogene
- D-GENE functionnal
- D-GENE ORF
- D-GENE pseudogene
- C-GENE functionnal
- C-GENE pseudogene
- RPI pseudogene

Supp figure 1: Western lowland gorilla (*Gorilla gorilla gorilla*) IGH locus on chromosome 14 assembly Kamilah_GGO_v0

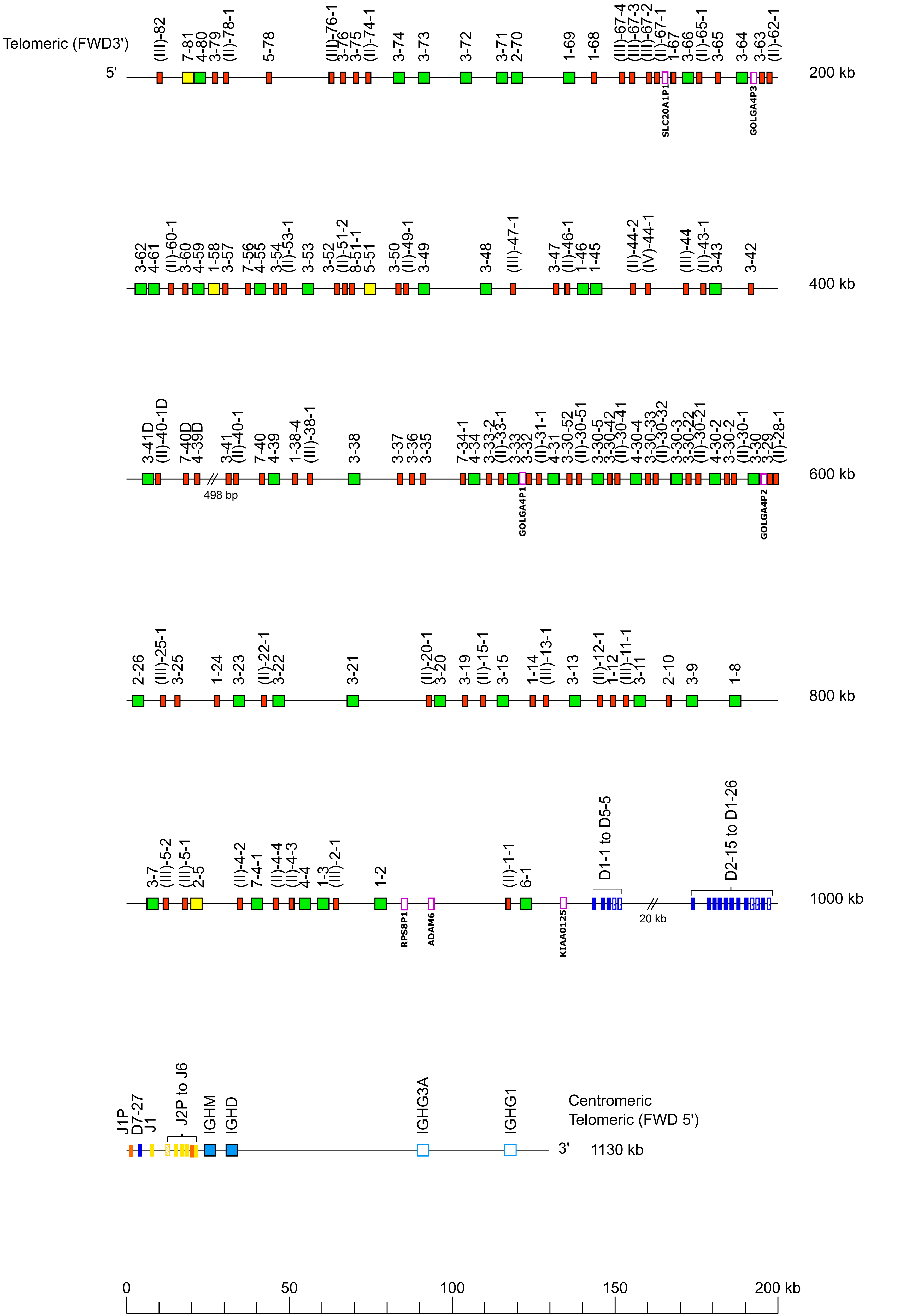

Supp figure 2: Western lowland gorilla (*Gorilla gorilla gorilla*) IGH locus on chromosome 14 assembly Susie3

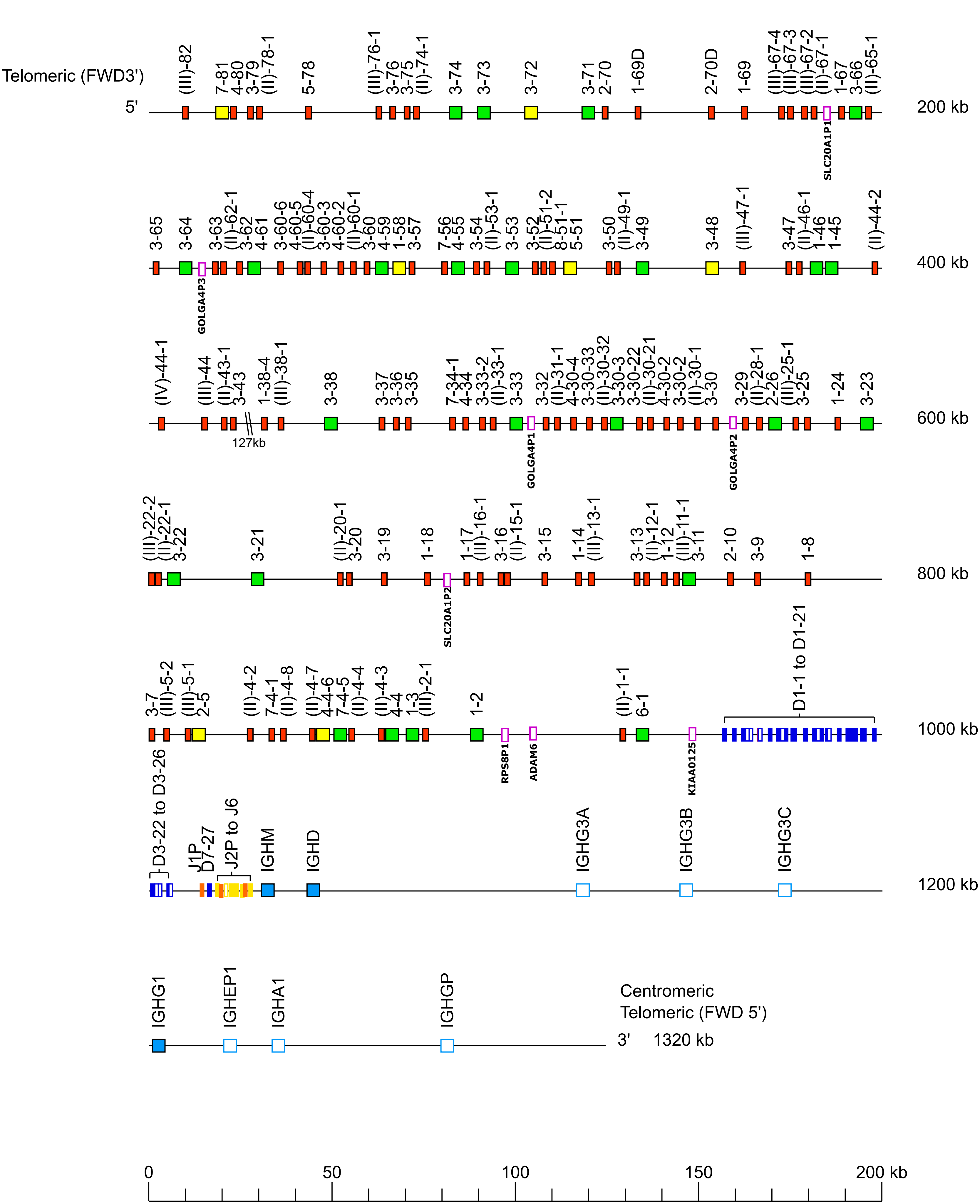

Supp figure 3: Western lowland gorilla (*Gorilla gorilla gorilla*) IGH locus on chromosome 14 assembly NHGRI_mGorGor1-v1.1-0.2.freeze_mat

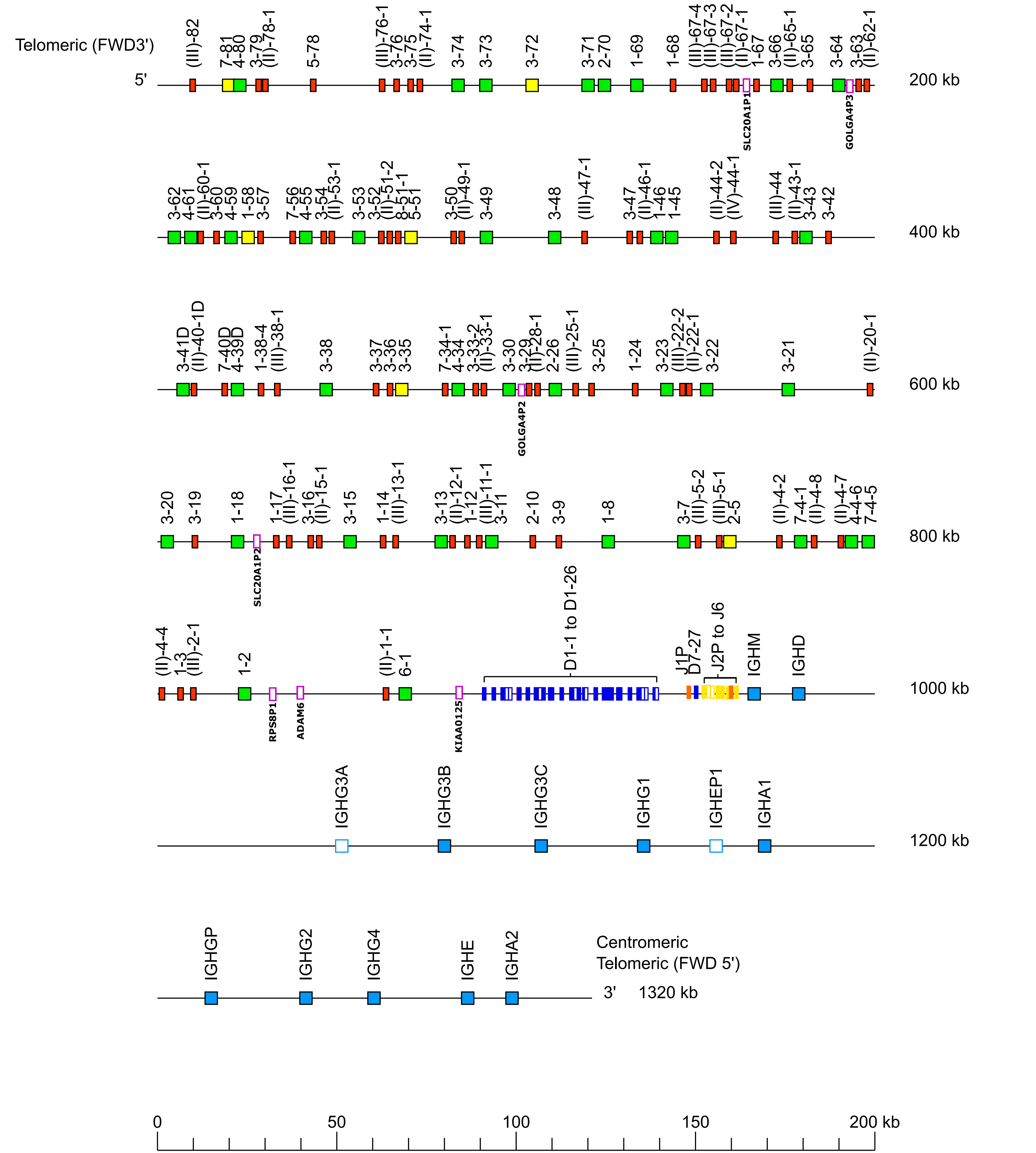

Supp figure 4: Western lowland gorilla (*Gorilla gorilla gorilla*) IGH locus on chromosome 14 assembly NHGRI_mGorGor1-v1.1-0.2.freeze_pat

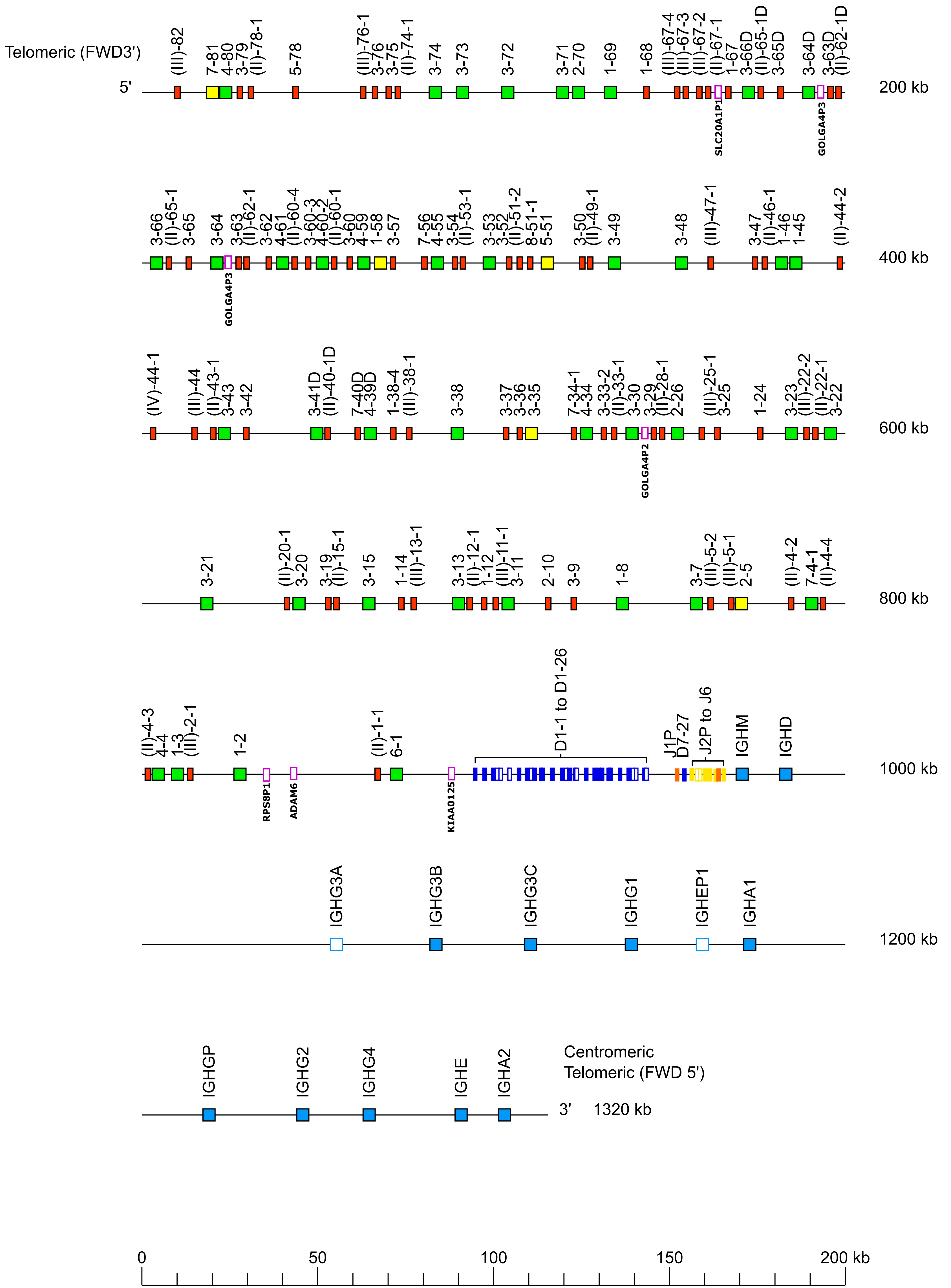

Supplement: Supplementary file 1 [file DataSheet1.zip › Supplementary_Material/Supplementary_figures_1_2_3_4.pdf]
