# Supplementary material for: Deciphering Gorilla gorilla gorilla immunoglobulin loci in multiple genome assemblies and enrichment of IMGT resources
Source: Front Immunol. 2024 Oct 10;15:1475003. doi: 10.3389/fimmu.2024.1475003 (PMC11499206; doi:10.3389/fimmu.2024.1475003)

Legend

- V-GENE functionnal
- V-GENE ORF
- V-GENE pseudogene
- J-GENE functionnal
- J-GENE ORF
- C-GENE functionnal
- C-GENE pseudogene
- RPI pseudogene
- 5' & 3' IMGT_borne gene functionnal

Supp figure 9: Western lowland gorilla (*Gorilla gorilla gorilla*) IGL locus on chromosome 22 assembly Kamilah_GGO_v0

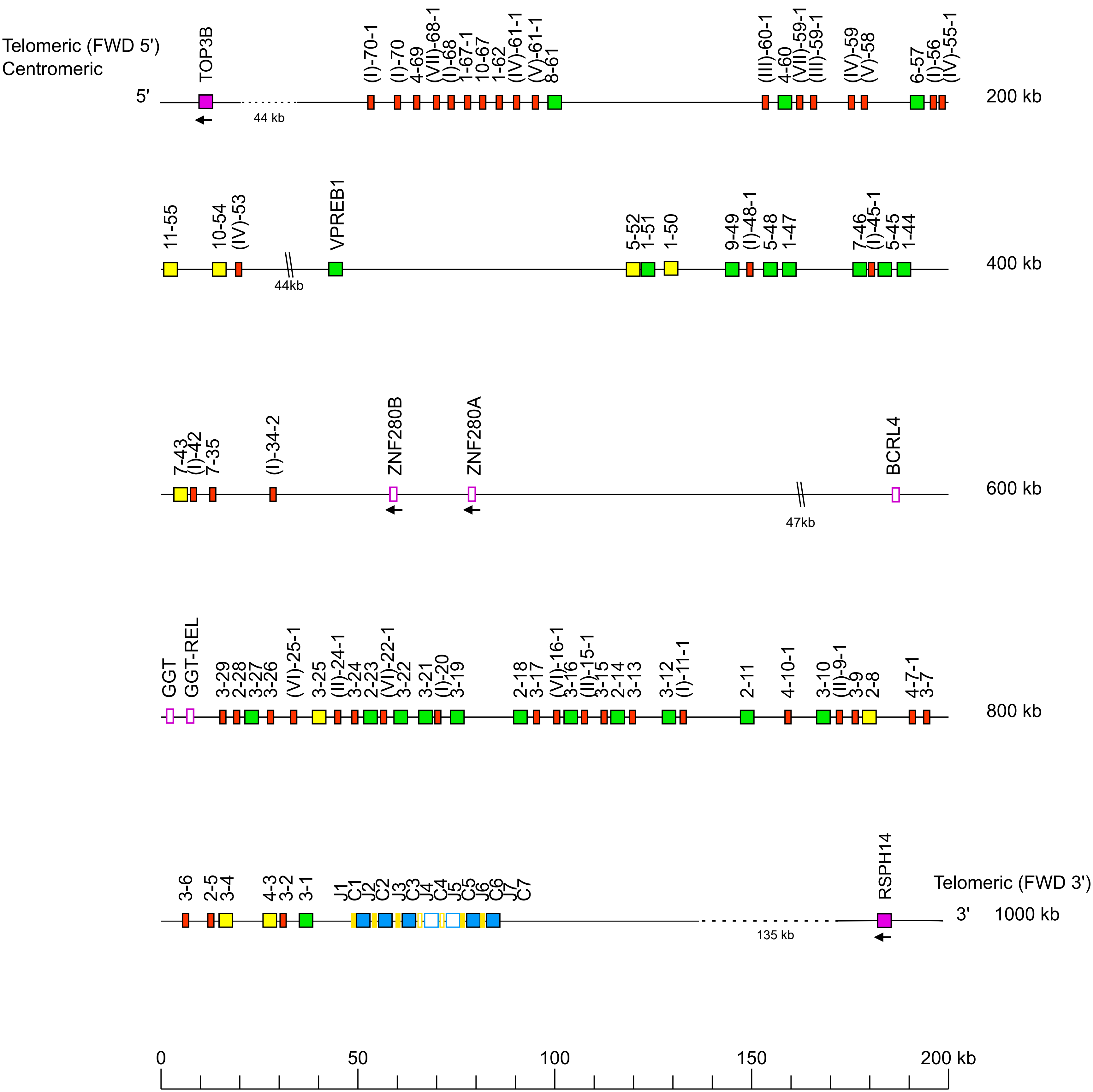

Supp figure 10: Western lowland gorilla (*Gorilla gorilla gorilla*) IGL locus on chromosome 22 assembly Susie3

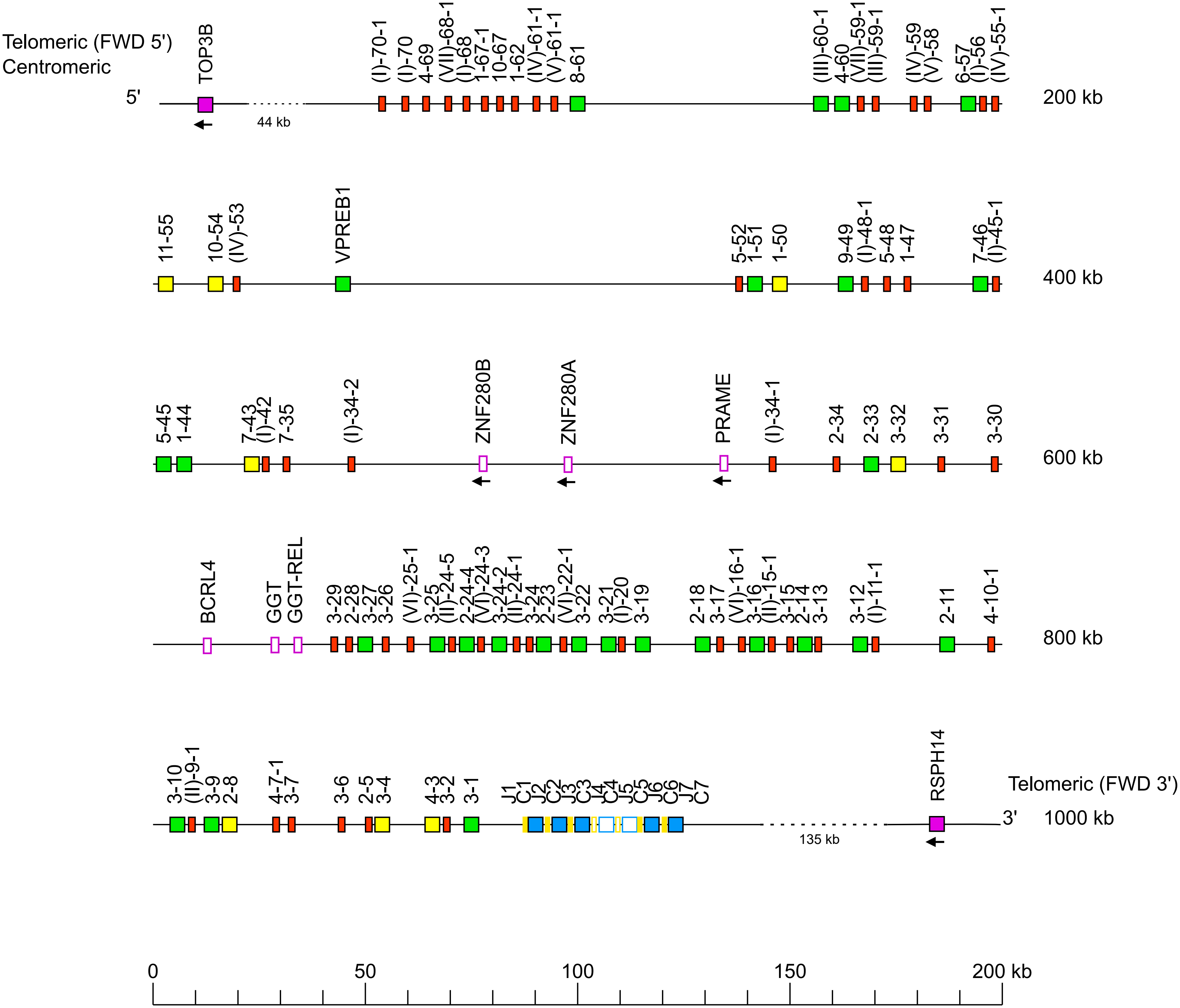

Supp figure 11: Western lowland gorilla (*Gorilla gorilla gorilla*) IGL locus on chromosome 22 assembly NHGRI_mGorGor1-v1.1-0.2.freeze_mat

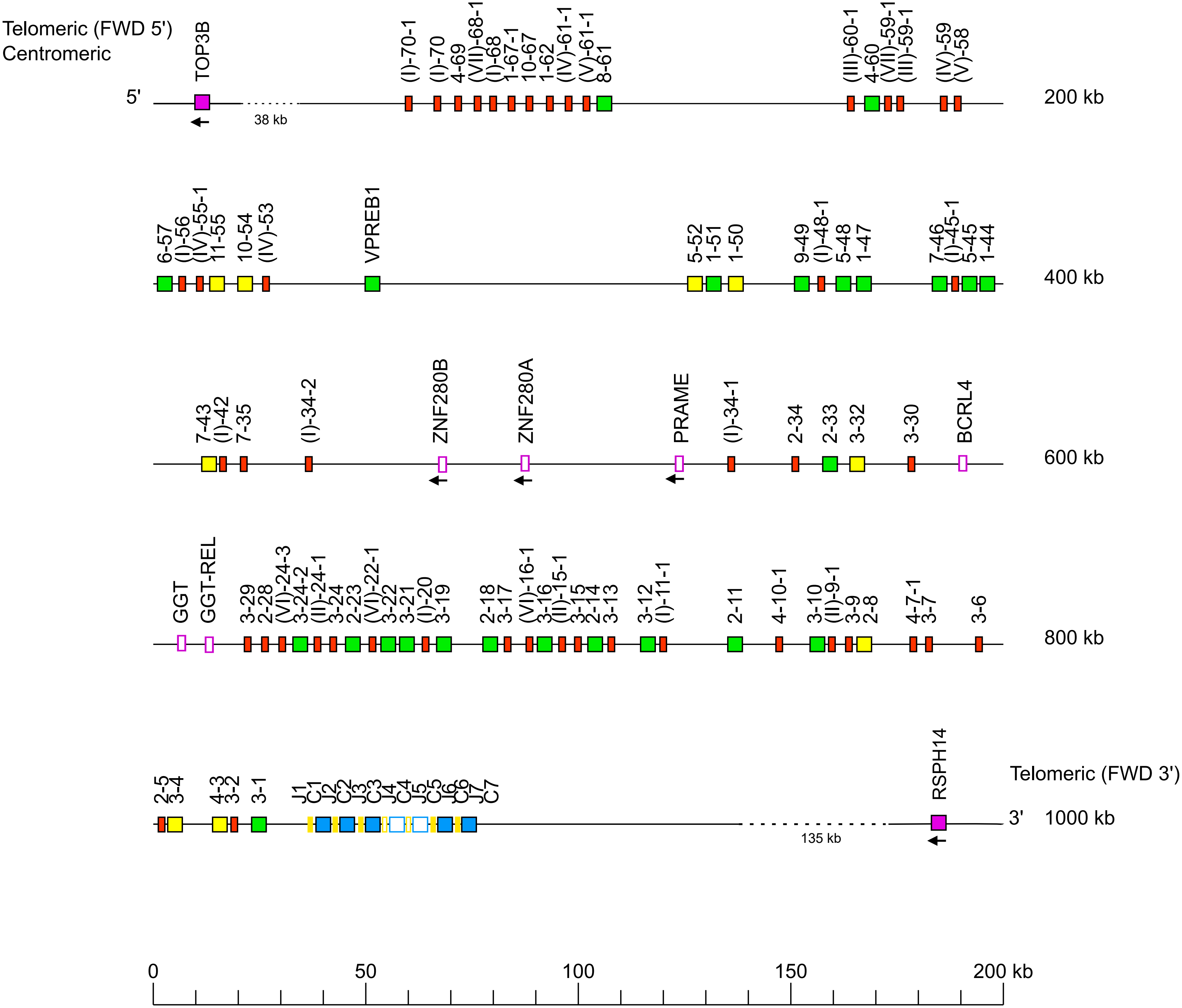

Supp figure 12: Western lowland gorilla (*Gorilla gorilla gorilla*) IGL locus on chromosome 22 assembly NHGRI_mGorGor1-v1.1-0.2.freeze_pat

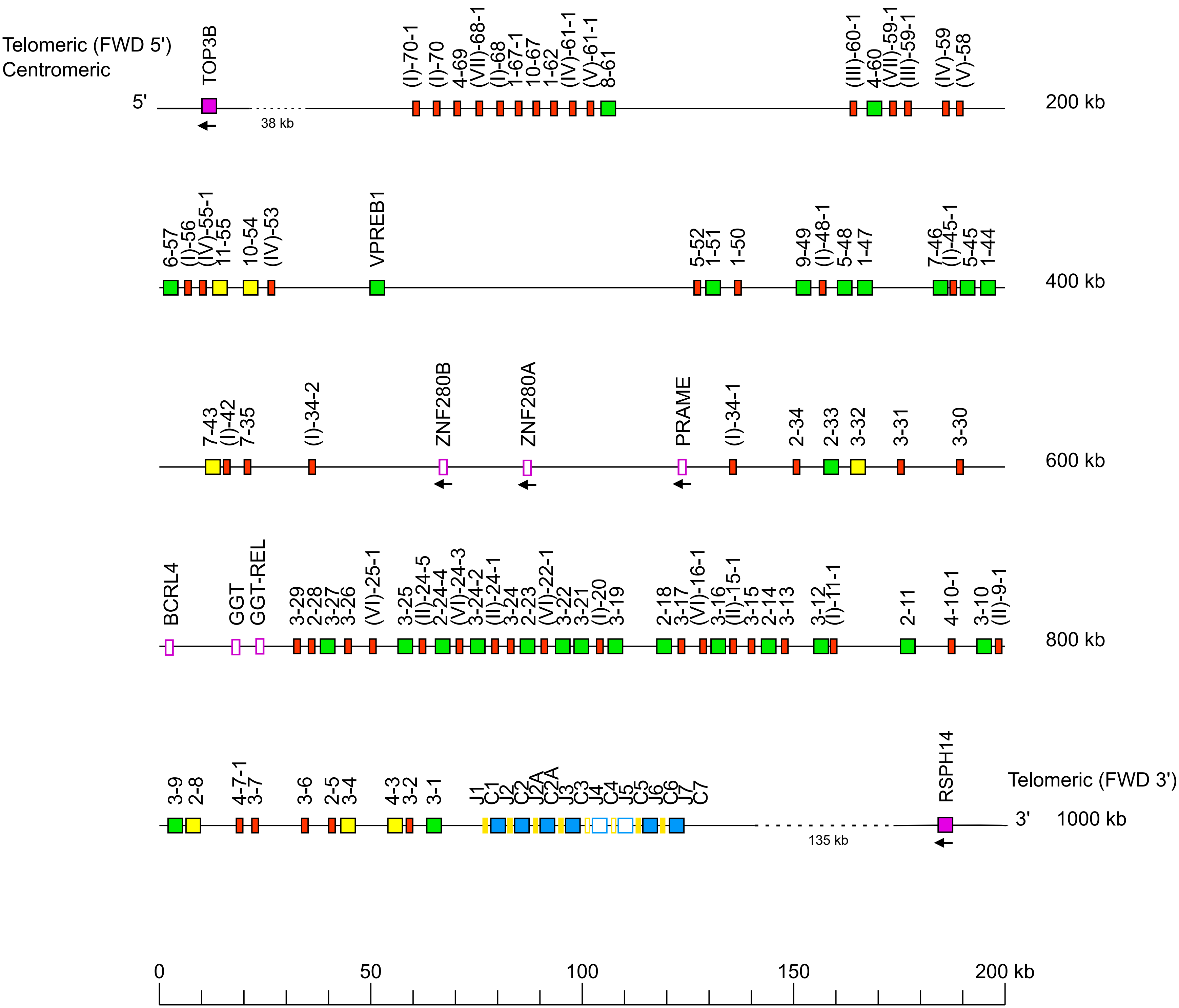

Supplement: Supplementary file 1 [file DataSheet1.zip › Supplementary_Material/Supplementary_figures_9_10_11_12.pdf]
