# Supplementary material for: Deciphering Gorilla gorilla gorilla immunoglobulin loci in multiple genome assemblies and enrichment of IMGT resources
Source: Front Immunol. 2024 Oct 10;15:1475003. doi: 10.3389/fimmu.2024.1475003 (PMC11499206; doi:10.3389/fimmu.2024.1475003)

# Supp figure 13: Phylogenetic tree of all IGK subgroups for *Gorilla gorilla gorilla* and *Homo sapiens*

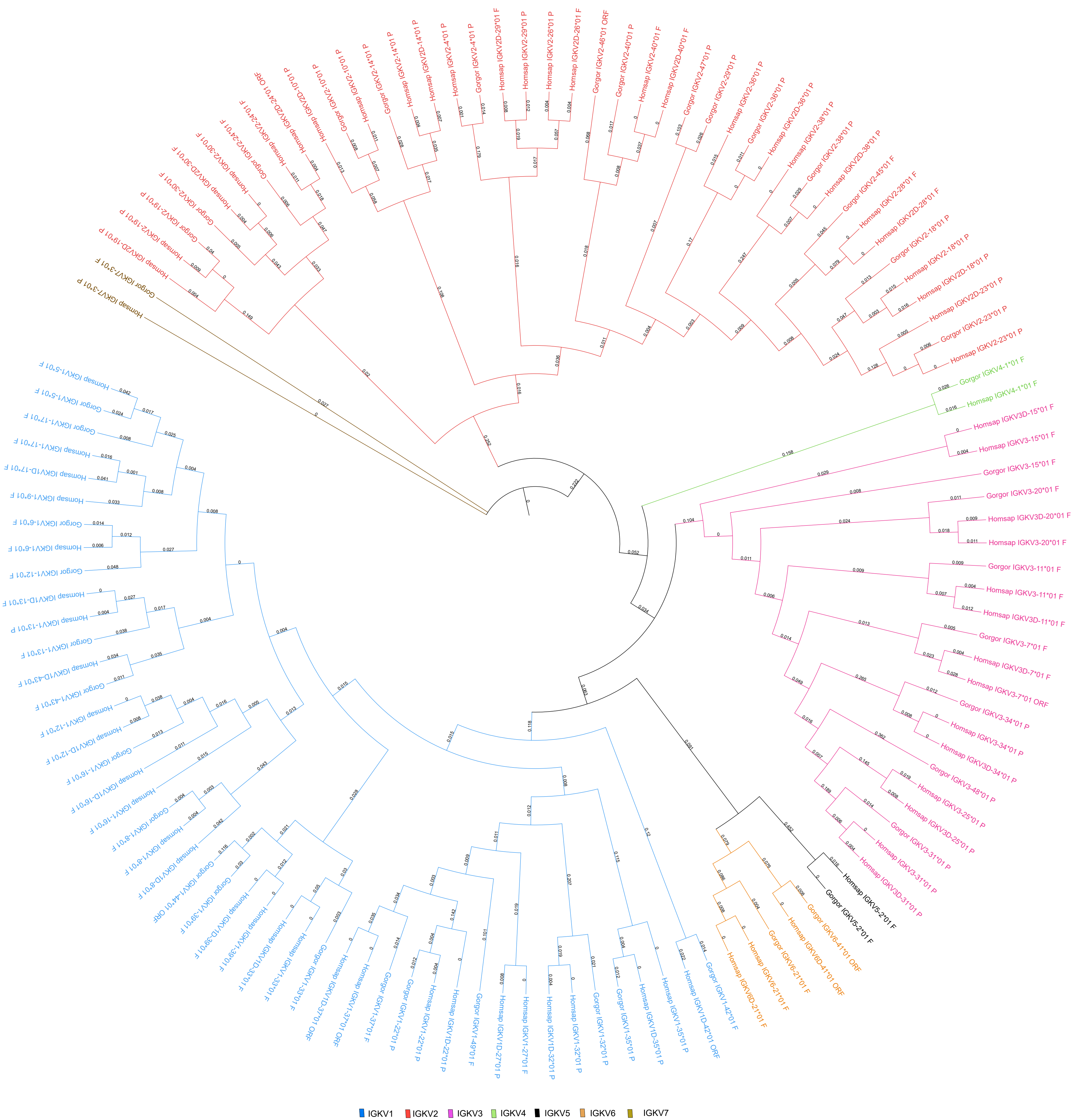

Supplement: Supplementary file 1 [file DataSheet1.zip › Supplementary_Material/Supplementary_figure_13_IGKV_phylogenetic_tree_for_human_&_gorilla.pdf]
