# Supplementary material for: Deciphering Gorilla gorilla gorilla immunoglobulin loci in multiple genome assemblies and enrichment of IMGT resources
Source: Front Immunol. 2024 Oct 10;15:1475003. doi: 10.3389/fimmu.2024.1475003 (PMC11499206; doi:10.3389/fimmu.2024.1475003)
Supplement: Supplementary file 1 [file DataSheet1.zip › Supplementary_Material/Supplementary_tables_5_6_7.pdf]

Supplementary table 5 numbers of Western lowland gorilla (*Gorilla gorilla gorilla*) functional F, Open reading frame O, pseudogene P IGHV gene per subgroup in IMGT annotated assemblies

IMGT Subgroup	Kamilah_GGO_v0		Susie3		NHGRI_mGorGor1-v1.1-0.2.freeze_mat		NHGRI_mGorGor1-v1.1-0.2.freeze_pat	
	**Gene nb**	**Total**	**Gene nb**	**Total**	**Gene nb**	**Total**	**Gene nb**	**Total**
IGHV1	6 F + 1 O + 6 P	13	4 F + 1 O + 10 P	15	6 F + 1 O + 8 P	15	6 F + 1 O + 6 P	13
IGHV2	2 F + 1 O + 1 P	4	1 F + 1 O + 3 P	5	2 F + 1 O + 1 P	4	2 F + 1 O + 1 P	4
IGHV3	26 F + 26 P	52	14 F + 2 O + 33 P	49	21 F + 2 O + 20 P	43	23 F + 1 O + 23 P	47
IGHV4	10 F + 1 P	11	6 F + 1 O + 4 P	11	7 F	7	8 F	8
IGHV5	1 O + 1 P	2	1 O + 1 P	2	1 O + 1 P	2	1 O + 1 P	2
IGHV6	1 F	1	1 F	1	1 F	1	1 F	1
IGHV7	1 F + 1 O + 4 P	6	1 F + 1 O + 3 P	5	2 F + 1 O + 3 P	6	1 F + 1 O + 3 P	5
IGHV8	1 P	1	1 P	1	1 P	1	1 P	1
IGHV(II)	29 P	29	29 P	29	24 P	24	26 P	26
IGHV(III)	14 P	14	16 P	16	16 P	16	15 P	15
IGHV(IV)	1 P	1	1 P	1	1 P	1	1 P	1
Total number of IMGT genes	46 F + 4 O + 84 P	134	27 F + 7 O + 101 P	135	39 F + 6 O + 75 P	120	41 F + 5 O + 77 P	123

Supplementary table 6 numbers of Western lowland gorilla (*Gorilla gorilla gorilla*) functional F, Open reading frame O, pseudogene P IGKV gene per subgroup in IMGT annotated assemblies

IMGT Subgroup	Kamilah_GGO_v0		Susie3		NHGRI_mGorGor1-v1.1-0.2.freeze_mat		NHGRI_mGorGor1-v1.1-0.2.freeze_pat	
	**Gene nb**	**Total**	**Gene nb**	**Total**	**Gene nb**	**Total**	**Gene nb**	**Total**
IGKV1	12 F + 1 O + 3 P	16	10 F + 6 P	16	13 F + 1 O + 3 P	17	12 F + 1 O + 3 P	16
IGKV2	3 F + 1 O + 11 P	15	2 F + 1 O + 10 P	13	4 F + 1 O + 10 P	15	4 F + 1 O + 10 P	15
IGKV3	4 F + 3 P	7	4 F + 3 P	7	4 F + 3 P	7	4 F + 3 P	7
IGKV4	1 F	1	1 P	1	1 F	1	1 F	1
IGKV5	1 F	1	1 F	1	1 F	1	1 F	1
IGKV6	1 F + 1 O	2	1 F + 1 O	2	1 F + 1 O	2	1 F + 1 O	2
IGKV7	1 F	1	1 F	1	1 F	1	1 P	1
Total number of IMGT genes	23 F + 3 O + 17 P	43	19 F + 2 O + 20 P	41	25 F + 3 O + 16 P	44	23 F + 3 O + 17 P	43

Supplementary table 7 numbers of Western lowland gorilla (*Gorilla gorilla gorilla*) functional F, Open reading frame O, pseudogene P IGLV gene per subgroup in IMGT annotated assemblies

IMGT Subgroup	Kamilah_GGO_v0		Susie3		NHGRI_mGorGor1-v1.1-0.2.freeze_mat		NHGRI_mGorGor1-v1.1-0.2.freeze_pat	
	Gene nb	Total	Gene nb	Total	Gene nb	Total	Gene nb	Total
IGLV1	3 F + 1 O + 2 P	6	2 F + 1 O + 3 P	6	3 F + 1 O + 2 P	6	3 F + 3 P	6
IGLV2	4 F + 1 O + 2 P	7	6 F + 1 O + 3 P	10	5 F + 1 O + 3 P	9	6 F + 1 O + 3 P	10
IGLV3	8 F + 2 O + 10 P	20	11 F + 2 O + 11 P	24	8 F + 2 O + 10 P	20	11 F + 2 O + 11 P	24
IGLV4	1 F + 1 O + 3 P	5	1 F + 1 O + 3 P	5	1 F + 1 O + 3 P	5	1 F + 1 O + 3 P	5
IGLV5	2 F + 1 O	3	1 F + 2 P	3	2 F + 1 O	3	2 F + 1 P	3
IGLV6	1 F	1	1 F	1	1 F	1	1 F	1
IGLV7	1 F + 1 O + 1 P	3	1 F + 1 O + 1 P	3	1 F + 1 O + 1 P	3	1 F + 1 O + 1 P	3
IGLV8	1 F	1	1 F	1	1 F	1	1 F	1
IGLV9	1 F	1	1 F	1	1 F	1	1 F	1
IGLV10	1 O + 1 P	2	1 O + 1 P	2	1 O + 1 P	2	1 O + 1 P	2
IGLV11	1 O	1	1 O	1	1 O	1	1 O	1
IGLV(I)	10 P	10	11 P	11	11 P	11	11 P	11
IGLV(II)	3 P	3	4 P	4	3 P	3	4 P	4
IGLV(III)	2 P	2	2 P	2	2 P	2	2 P	2
IGLV(IV)	4 P	4	4 P	4	4 P	4	4 P	4
IGLV(V)	2 P	2	2 P	2	2 P	2	2 P	2
IGLV(VI)	3 P	3	4 P	4	3 P	3	4 P	4
IGLV(VII)	2 P	2	2 P	2	2 P	2	2 P	2
Total number of IMGT genes	22 F + 9 O + 45 P	76	25 F + 8 O + 53 P	86	23 F + 9 O + 47 P	79	27 F + 7 O + 52 P	86
